# AI generations: from AI 1.0 to AI 4.0

**DOI:** 10.3389/frai.2025.1585629

**Published:** 2025-06-26

**Authors:** Jiahao Wu, Hengxu You, Jing Du

**Affiliations:** Informatics, Cobots and Intelligent Construction Lab, Engineering School of Sustainable Infrastructure and Environment, University of Florida, Gainesville, FL, United States

**Keywords:** artificial intelligence evolution, machine learning, reinforcement learning, large language models, AI ethics and governance

## Abstract

This paper proposes that Artificial Intelligence (AI) progresses through several overlapping generations: AI 1.0 (Information AI), AI 2.0 (Agentic AI), AI 3.0 (Physical AI), and a speculative AI 4.0 (Conscious AI). Each AI generation is driven by shifting priorities among algorithms, computing power, and data. AI 1.0 accompanied breakthroughs in pattern recognition and information processing, fueling advances in computer vision, natural language processing, and recommendation systems. AI 2.0 is built on these foundations through real-time decision-making in digital environments, leveraging reinforcement learning and adaptive planning for agentic AI applications. AI 3.0 extended intelligence into physical contexts, integrating robotics, autonomous vehicles, and sensor-fused control systems to act in uncertain real-world settings. Building on these developments, the proposed AI 4.0 puts forward the bold vision of self-directed AI capable of setting its own goals, orchestrating complex training regimens, and possibly exhibiting elements of machine consciousness. This paper traces the historical foundations of AI across roughly 70 years, mapping how changes in technological bottlenecks from algorithmic innovation to high-performance computing to specialized data have stimulated each generational leap. It further highlights the ongoing synergies among AI 1.0, 2.0, 3.0, and 4.0, and explores the ethical, regulatory, and philosophical challenges that arise when artificial systems approach (or aspire to) human-like autonomy. Ultimately, understanding these evolutions and their interdependencies is pivotal for guiding future research, crafting responsible governance, and ensuring that AI’s transformative potential benefits society.

## 1 Introduction

Artificial Intelligence (AI) has experienced a transformative evolution over the last 70 years, evolving from its nascent stage of theoretical formulations to its current status as a cornerstone of technological advancement (Haenlein and Kaplan, 2019). Initially, the field was dominated by intellectual explorations into symbolic reasoning, knowledge representation, and the rudimentary principles of machine learning (Newell and Simon, 1956). These early stages were marked by a focus on conceptual breakthroughs, laying the groundwork for what AI could potentially achieve. As computational capabilities expanded and data sources proliferated, AI transitioned from theoretical models to practical applications capable of learning from patterns and making precise predictions (Alom et al., 2018). The last two decades, however, have witnessed an unprecedented acceleration in AI development, propelling the field into realms that surpass even the most optimistic projections of its early pioneers.

Despite remarkable successes in areas like natural language processing, computer vision, and large-scale data analytics, AI continues to face challenges in interacting seamlessly with complex, dynamic real-world environments. This ongoing struggle signals an emerging phase in AI’s evolution, marking a shift from systems that primarily process and predict information to ones that can plan, decide, and act, ushering in new generations of AI: *Information AI (AI 1.0)*, *Agentic AI (AI 2.0)*, *Physical AI (AI 3.0)* and *Conscious AI (AI 4.0)*. This classification not only clarifies the conceptual transitions within the field but also helps delineate the evolution of AI capabilities from data extraction to making autonomous decisions in digital realms, and now to engaging directly with the physical world.

Understanding these transitions is essential, not just from a technological standpoint but also for grasping the societal and economic implications of AI. Distinct technological drivers and bottlenecks have shaped each phase of AI: the early period was limited by the lack of advanced algorithms and computational frameworks (Jones, 1994); the advent of powerful GPUs around 2012 significantly shifted the landscape, enabling more complex neural architectures (Nvidia, 2011); and today, the challenge has moved toward harnessing domain-specific, high-quality data to feed into these sophisticated systems (Budach et al., 2022). Recognizing these shifts is crucial for stakeholders, including policymakers, researchers, and industry leaders, who must navigate the ethical, regulatory, and technical complexities introduced by advanced AI systems.

This review aims to provide a comprehensive retrospective on the milestones that have defined AI’s progress. By tracing the lineage of algorithmic innovations, increases in computing power, and enhancements in data utilization, we aim to illuminate the significant moments that have shaped AI from its inception to its current state. This exploration is structured around the AI 1.0 to AI 4.0 framework, illustrating how each generation’s defining features and limitations correspond to broader historical phases from approximately 1950 to the present. In doing so, we will also contemplate the future trajectory of AI, considering the potential technical challenges, societal impacts, and strategic directions that could define the next phases of AI research and application.

This article is structured first to revisit the historical foundations of AI, emphasizing the shifts in primary drivers from algorithms to computing power to data. We then delve into the specific characteristics, achievements, and limitations of AI 1.0, AI 2.0, AI 3.0, and AI 4.0. Following this, we explore AI’s convergence and future outlook, highlighting the synergies among the four generations and outlining the grand challenges that lie ahead. Finally, we conclude with a synthesis of key insights and propose future directions for sustained progress in the field, aiming to both inform and inspire continued innovation and thoughtful integration of AI into our daily lives and societal structures.

## 2 Historical foundations of AI

### 2.1 Phase 1 (1950s–2010s): Age of algorithmic innovations

Since the 1950s, AI has advanced through a dynamic interplay among three core ingredients: *algorithms*, *computing power*, and *data* (Schmidhuber, 2022). Although these three factors have always shaped the field, they have not always contributed equally at every stage. In the early decades, the limiting factor was innovation in algorithms. From mid-century debates about the feasibility of machine intelligence to the emergence of expert systems and neural networks, it was clear that conceptual breakthroughs would determine AI’s boundaries (Turing, 2009). Meanwhile, although data and computing power were important, they played more supportive roles. Gradually, as new hardware architectures appeared and large-scale datasets became more accessible, the focus shifted toward harnessing immense computational capability and vast amounts of information. During this era, most funding for algorithmic research came from government programs (e.g., DARPA’s Strategic Computing Initiative) and a handful of industrial labs, fostering tight collaborations between computer scientists, control engineers, and cognitive psychologists to maximize limited hardware through smarter algorithms.

From the outset, researchers were fascinated by whether machines could truly think. Alan Turing’s pioneering paper (Turing, 1950) set the stage, posing the famous “*imitation game*” as a litmus test for intelligence. In 1956, the Dartmouth Conference (McCarthy et al., 2006) formally introduced the term “Artificial Intelligence” and laid out the bold proposition that the essence of human intelligence could be precisely described and replicated in machines. Early NSF and DARPA grants enabled interdisciplinary AI centers at MIT and Stanford, where mathematicians, linguists, and early cybernetics experts worked side by side to turn the Turing Test and Dartmouth vision into functioning prototype systems. Early systems, such as the *Logic Theorist* and the *General Problem Solver* (Newell and Simon, 1956; Newell et al., 1959) underscored that symbolic reasoning could be computationally realized. These proof-of-concept attempts highlighted the central premise of that era: if we could devise the right algorithms, computers might reason and solve problems with near-human efficacy.

By the 1960s and 1970s, a strong emphasis on *symbolic AI* emerged. Influential works by McCarthy (1960) introduced LISP as a language suited to symbolic processing, while Minsky and Papert’s (1969) critical analysis of single-layer perceptions contributed to a pause in neural network research, pushing many researchers toward knowledge-based or “expert” systems. Milestones like the DENDRAL project (Buchanan et al., 1971) and MYCIN (Shortliffe et al., 1975) showcased how carefully curated rule sets could guide problem-solving in specialized domains. These systems illustrated the power of algorithmic design in areas such as medical diagnosis or chemical analysis, even when real-world data were scarce and computational resources were limited. Corporate and university partnerships in domains like healthcare (e.g., with Stanford Medical School) and chemical analysis funded expert-system projects, creating joint labs where domain specialists and AI researchers codified knowledge bases despite constrained memory and CPU budgets.

**Figure 1:**
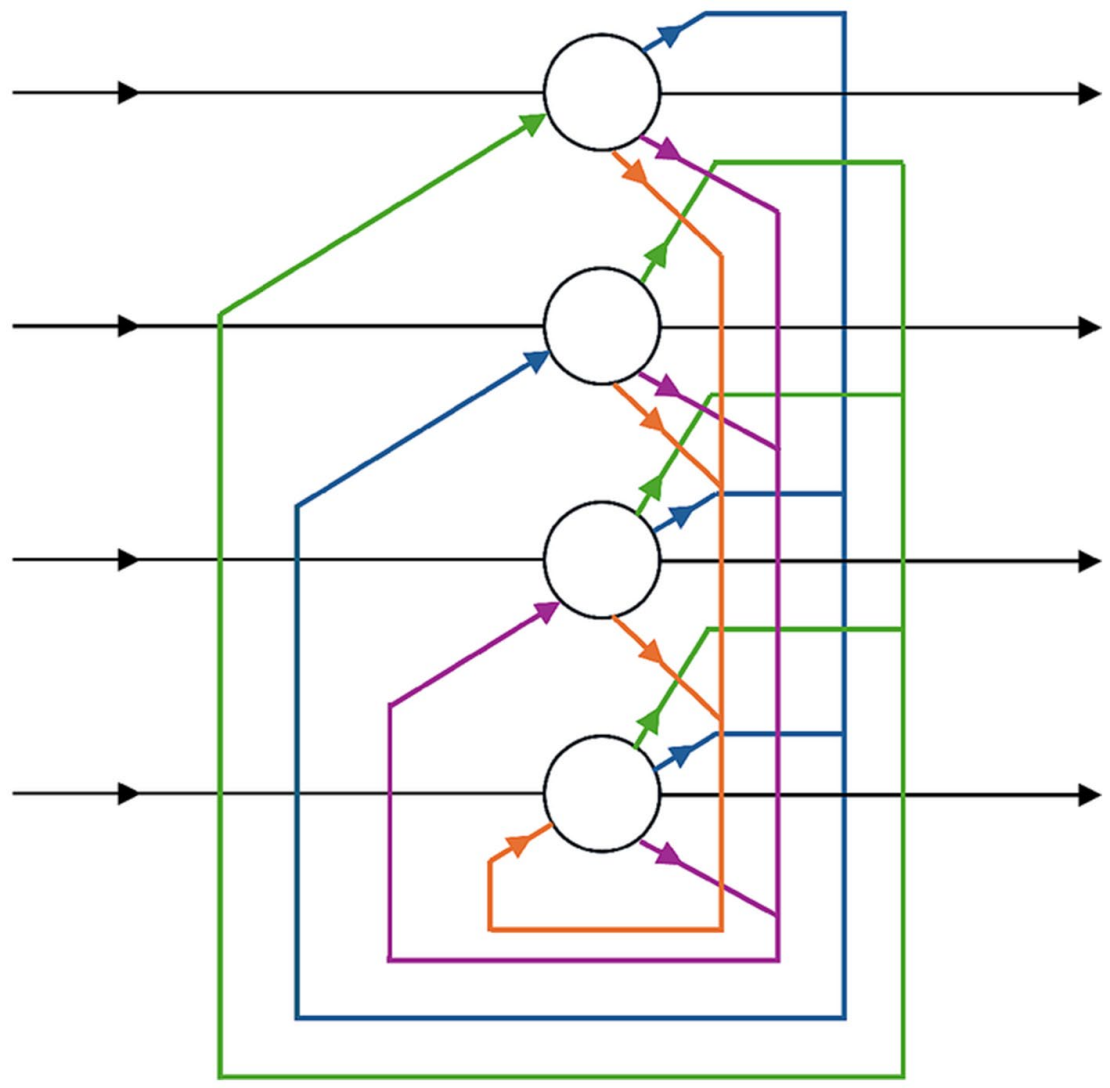
The Hopfield networks (Hopfield, 1982) introduced content-addressable memory in neural networks, marking a major milestone in connectionism in AI.

Neural networks rebounded in the 1980s with work on Hopfield networks (Hopfield, 1982) (Figure 1) and, crucially, the rediscovery of backpropagation (Rumelhart et al., 1986). This gave researchers fresh insight into how machines might learn patterns from data. Though the potential of these connectionist approaches was clear, they often stalled because large datasets were not widely available and specialized hardware did not yet exist. Even so, foundational contributions like LeCun et al. (1989) application of *convolutional neural networks* to handwritten digit recognition laid the groundwork for modern deep learning. Modest government programs and early industry prototypes, such as the Connection Machine, emerged from collaborations between neuroscientists and computer engineers. Still, widespread adoption had to await later GPU cost declines and cloud-computing services.

**Figure 2:**
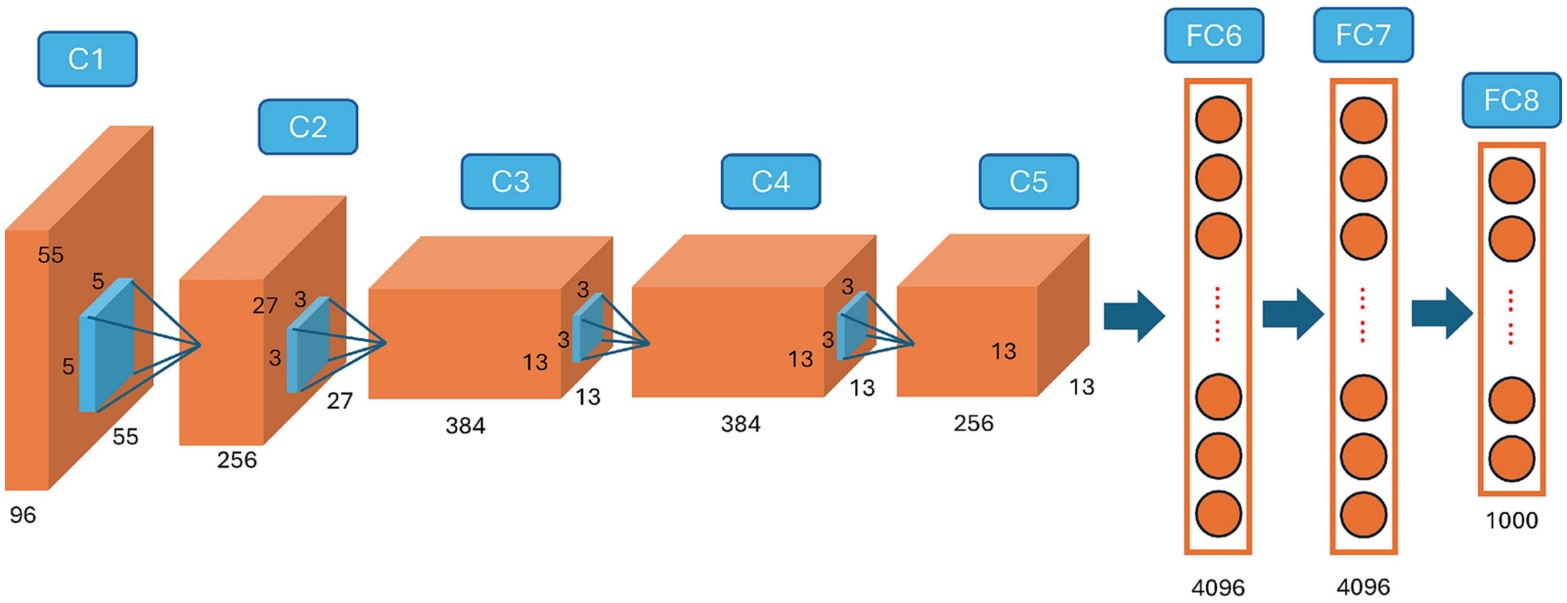
AlexNet (Krizhevsky et al., 2012) marks the beginning of large-scale, GPU-accelerated convolutional neural networks for high-performance image classification.

By the 1990s, specific algorithmic achievements hinted at deeper architectures capable of tackling increasingly complex tasks. The proposal of Long Short-Term Memory (LSTM) networks effectively addressed the vanishing gradient problem, opening possibilities for modeling sequential data more accurately (Hochreiter, 1997). However, the real transformative moment emerged around 2012, when Krizhevsky, Sutskever, and Hinton demonstrated that ImageNet-scale datasets and high-performance GPUs could dramatically improve a deep neural network’s ability to classify images, i.e., the *AlexNet* (Krizhevsky et al., 2012) (Figure 2). Although this watershed event is often viewed as the dawn of the “deep learning era,” it could not have happened without the algorithmic groundwork laid over the preceding decades. The 2012 AlexNet breakthrough itself was propelled by the ImageNet consortium, uniting academic vision labs and industry hardware vendors, and by the sudden availability of affordable GPU clusters donated or subsidized by major tech companies.

### 2.2 Phase 2 (2010s–present): The computing revolution and deep learning renaissance

The pattern-matching architectures pioneered in AI 1.0, such as convolutional filters for edge and shape detection and Hopfield networks for associative memory, laid the essential groundwork for AI 2.0’s learned feature hierarchies. By encoding low and mid-level visual and sequential patterns in trainable layers, these early connectionist models enabled decision-making agents to operate on rich, automatically extracted representations rather than raw sensor data, accelerating reinforcement-learning and supervised learning breakthroughs once sufficient data and compute became available.

**Figure 3:**
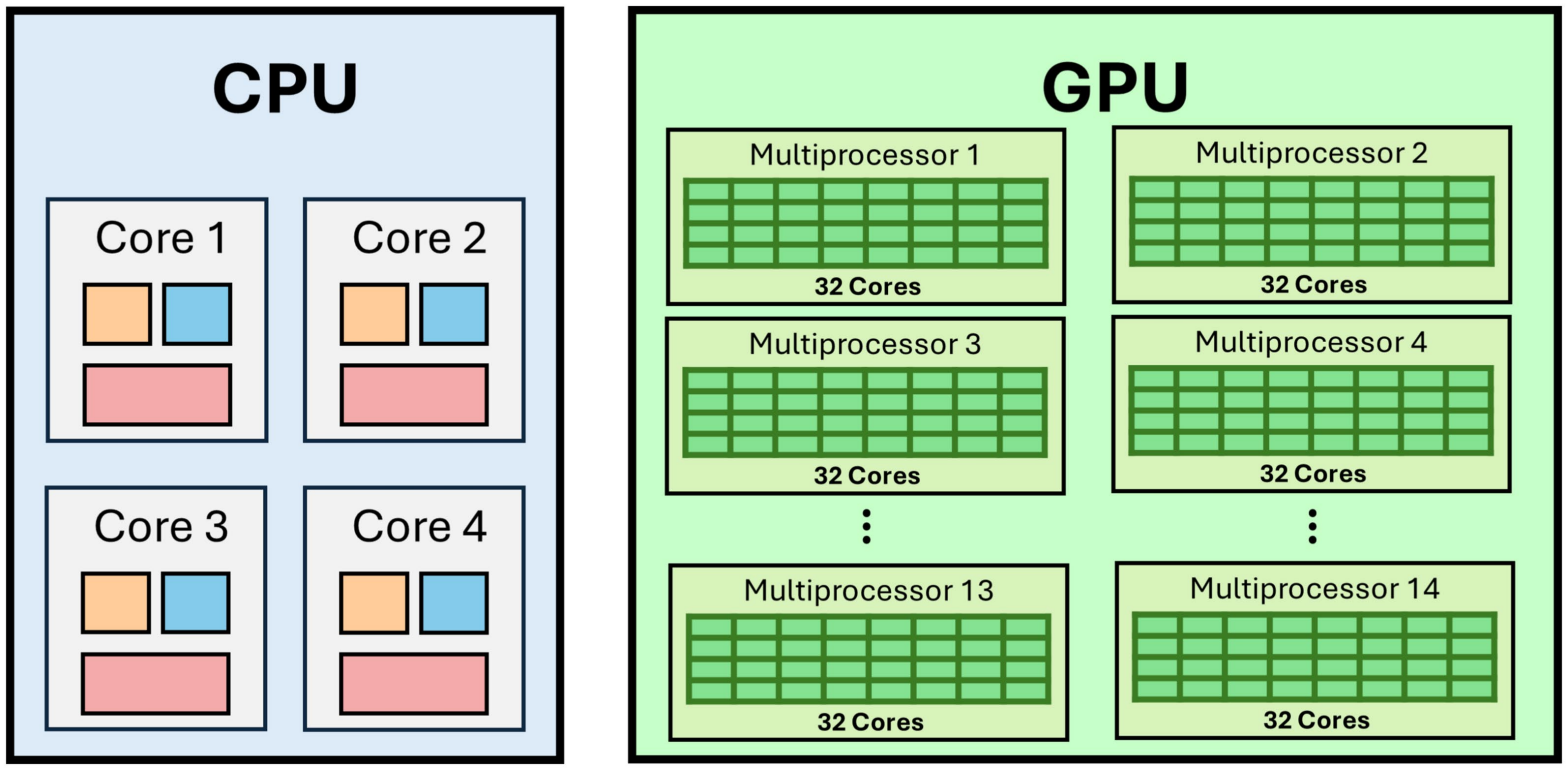
The CUDA architecture pioneered general-purpose GPU computing, revolutionizing parallel processing and accelerating AI breakthroughs.

A dramatic shift in AI research took hold around 2012, when mounting computational capacity began to eclipse algorithmic novelty as the principal engine of progress. This transition was underwritten by rapidly declining GPU prices, driven by consumer gaming markets, and by major cloud providers (AWS, Google Cloud, Azure) offering GPU instances, which democratized access to parallel computing. Key partnerships between hardware vendors (NVIDIA, AMD) and academic labs established early benchmarks for large-scale training, exemplifying how economic incentives catalyze scientific breakthroughs. While the core concepts underlying neural networks had been present since at least the 1980s, it was the widespread adoption of General-Purpose Graphics Processing Units (GPUs) that ignited what is often termed the “*deep learning renaissance*” (Figure 3) (Nickolls et al., 2008). Collaborative consortia, such as the ImageNet project, brought together vision researchers, software engineers, and data curators from both academia and industry, creating shared data resources and open-source codebases that accelerated innovation and reproducibility. When Krizhevsky et al. (2012) leveraged GPUs to train a large convolutional neural network for the ImageNet competition, they decisively demonstrated how parallelized computing could unearth performance gains previously unachievable with single-threaded Central Processing Units (CPUs). This milestone was enabled by government grants and corporate research labs (e.g., Google Brain, Microsoft Research), which invested in GPU clusters and supported interdisciplinary teams of machine-learning scientists and systems engineers to push the limits of scale. This turning point catalyzed a wave of research across machine vision, speech recognition, and natural language processing, with groups at Google, Microsoft, Baidu, and many academic institutions all racing to scale up network architectures (Dean et al., 2012; Bishop, 2013; Yu, 2013). The essence of this period lay in the conviction that “bigger is better,” whether in terms of model parameters, dataset size, or sheer computational resources. Consequently, much of the state-of-the-art progress hinged on harnessing specialized hardware: first GPUs, then tensor processing units (TPUs) and other custom accelerators, to churn through ever-growing datasets in shorter training cycles.

By the mid-2010s, the explosive rise of deep reinforcement learning (Mnih et al., 2015) and breakthroughs in game-playing AI, such as AlphaGo (Silver et al., 2016), underscored that not only could AI models learn representations from massive data, but they could also discover winning strategies through large-scale simulations. These advances were propelled by collaborations between AI theorists, neuroscientists studying decision-making, and high-performance computing experts, as well as by significant venture-capital funding in AI startups focusing on simulation-based learning and autonomous agents. Nevertheless, the predominant realm for these systems remained resolutely digital. Whether classifying images, translating text (Bahdanau, 2014; Vaswani, 2017), or playing complex board and video games, AI was still operating in an essentially informational context. Although data availability was critical and algorithms like convolutional and recurrent neural networks continued to improve, sheer computational power was often the deciding factor in achieving superior performance. Researchers observed emergent patterns in “*scaling laws*” (Kaplan et al., 2020), revealing that larger models trained on larger datasets could unlock qualitatively new capabilities. Systems like GPT-2 (Radford et al., 2019) and GPT-3 (Brown et al., 2020) illustrated this phenomenon vividly by demonstrating a striking ability to generate human-like text once parameter counts and training data reached certain thresholds. The development and deployment of these language models were driven by multi-institutional efforts, including OpenAI’s partnerships with cloud providers and academic collaborators, and by economic incentives from industries eager to commercialize natural-language interfaces, fueling research consortia around ethical and scalable model training. Because of their sophistication, these models continued to reside in the digital world, making them refined and powerful versions focused on big data analytics and pattern recognition at an unprecedented scale. Even so, the end of this phase began to hint at a transition toward greater autonomy and decision-making in digital contexts, an emerging hallmark of agentic AI. While many systems are still centered on classification or prediction, the rise of advanced reinforcement learning agents able to adapt strategies within software ecosystems foreshadowed a new kind of agency. By approximately 2024, the scholarly and commercial drive to develop goal-directed virtual assistants, automated resource allocation tools, and multi-agent simulations suggested that the chief challenge was no longer purely to label data accurately, but to act in digital environments in ways that transcended traditional supervised learning (Chen et al., 2023). This growing desire for agentic AI remained tied to abundant computing power, yet it began to reveal new dependencies on specialized data streams and real-time feedback loops (Tosi et al., 2024). It set the stage for the next generation of AI, where computational needs would remain vital. Still, data and context-specific knowledge would become even more pivotal in enabling truly autonomous, adaptive systems.

However, this unprecedented shift toward data-driven and compute-driven breakthroughs has also exposed systemic vulnerabilities that must be carefully examined before embracing the next wave of autonomous, agentic AI. The deployment of AI 2.0 into high-stakes domains such as finance, public policy, and healthcare has revealed how tightly coupled, speed-optimized systems can trigger cascading failures under stress. In financial markets, for instance, high-frequency trading algorithms, tuned to exploit sub-millisecond price discrepancies, precipitated the “Flash Crash” of May 6, 2010, when linked bots erased nearly $1 trillion in equity value within minutes before a partial rebound (Kirilenko et al., 2017). Without unified circuit breakers or oversight mechanisms, these agents amplified feedback loops during extreme volatility, demonstrating that raw performance can come at the price of systemic stability. In law enforcement, predictive-policing tools trained on decades of arrest data in Chicago and Los Angeles disproportionately targeted minority neighborhoods, perpetuating historical biases and eroding community trust. Credit-scoring models have likewise been shown to underprice loans for underrepresented groups, prompting regulatory investigations into discriminatory lending practices. These examples underscore that high accuracy on benchmark datasets does not guarantee equitable or safe outcomes in complex, real-world settings. Healthcare AI offers a further cautionary tale: diagnostic assistants trained on skewed image collections have misclassified critical conditions in underrepresented populations. A prominent 2018 study found that a melanoma detection model, trained predominantly on light-skinned images, misdiagnosed darker-skinned patients at twice the rate of lighter-skinned counterparts, despite reporting >95% accuracy on its test set. Such failures highlight the dangers of blindly scaling models without rigorous data curation and validation protocols. To guard against these risks, we recommend a multilayered defense: comprehensive stress testing under extreme or adversarial conditions; mandated transparency of model architectures and data provenance to facilitate third-party audits; regulatory circuit-breakers and human-in-the-loop overrides in mission-critical systems; and the formation of interdisciplinary oversight bodies that bring together AI practitioners, ethicists, domain experts, and policymakers. Embedding these safeguards will enable the AI community to harness the power of deep learning while preserving social, economic, and ethical stability.

### 2.3 Phase 3 (2024–foreseeable future): Data-centric paradigms

In the wake of a period defined by dramatic increases in computational horsepower, the focal point of AI advancement has shifted once again. This transition has been propelled by major industry investments, particularly from cloud providers (AWS, Google Cloud) offering specialized GPU/TPU instances, and by venture capital funding in data-centric startups, which drove large-scale data-aggregation platforms and new public-private consortia to curate domain-specific datasets. Where Phase 2 thrived on scaling neural networks through unprecedented parallel processing, Phase 3 acknowledges that data, especially specialized, high-quality data, is frequently the greatest obstacle. Researchers have discovered that ever-larger models alone do not guarantee success without context-rich training sets. Consequently, large-scale, domain-specific data-collection efforts have emerged, reshaping the field’s priorities. Projects that aggregate specialized medical data for diagnostic systems (Topol, 2019), simulate high-fidelity environments for robotics and autonomous vehicles (Dosovitskiy et al., 2017; Kalashnikov et al., 2018), or compile deep reinforcement learning benchmarks with realistic constraints (Bellemare et al., 2013; Dulac-Arnold et al., 2021) attest to the idea that harnessing robust datasets can be as determinative as algorithmic ingenuity or raw computational power.

Despite the continued importance of parallel computing and innovative architectures, many cutting-edge successes now hinge on data strategy. The “data-centric AI” movement gained traction through collaborations between academic labs (e.g., Stanford’s DAWN project) and industry partners in healthcare, automotive, and finance, where structured data pipelines and synthetic data initiatives (e.g., NVIDIA’s DRIVESim) received dedicated research grants and created shared benchmarks. Researchers have championed “data-centric AI” (Ng, 2021), arguing that refining training sets, removing biases, filling in coverage gaps, or generating synthetic data to handle edge cases, often yields more improvement than adding layers to a neural network. This philosophy is closely related to the rise of foundation models (Bommasani et al., 2021), which are vast neural architectures that can be adapted to myriad tasks but require massive, carefully curated corpora to realize their full potential. As data becomes the true bottleneck, teams must grapple with the logistical and ethical challenges of collecting, storing, and labeling it, as well as with privacy, consent, and representation issues.

Within this phase, AI’s transition from informational analysis to agentic decision-making becomes increasingly tangible. Interdisciplinary teams combining roboticists, control engineers, and ethicists, backed by government programs like the U. S. National Robotics Initiative and by multinational R&D labs (e.g., Toyota Research Institute), have spearheaded projects in autonomous vehicles, surgical robotics, and drone swarms, underscoring how robust data collection and simulation frameworks enable real-world agentic AI (Hicks and Simmons, 2019). Reinforcement learning agents not only plan and learn in complex digital worlds but also begin to bridge into real-world applications, where they must reason about noisy sensors, hardware uncertainties, and human collaboration. Physical AI, exemplified by advanced robotics, autonomous drones, and integrated cyber-physical systems, moves beyond the boundaries of simulated or purely informational spaces. However, this shift toward real-world, data-driven embodiments brings its own economic and logistical hurdles. High-precision sensors (LiDAR, RGB-D cameras, IMUs) and edge-grade compute (GPUs, FPGAs, TPUs) substantially increase hardware costs and power consumption, shortening operational endurance and increasing maintenance overhead. As teams move from single prototypes to fleet deployments, these expenses multiply and place heavy demands on network bandwidth for firmware updates and sensor recalibrations. Energy-efficiency constraints can limit mission duration in field robots and drones, making the economic trade-offs of embodied autonomy as critical to system design as algorithmic accuracy or robustness. Progress in robotic grasping and manipulation (Kalashnikov et al., 2018; Levine et al., 2018), self-driving vehicles (Bojarski, 2016), and robotic surgery (Yang et al., 2017) signals how these systems can robustly interact with the environment, handle dynamic conditions, and learn from continuous feedback. Thus, the hallmark of this new phase is the recognition that data unlocks the fuller potential of agentic AI in digital ecosystems, as well as physically embodied intelligence in the real world (Fiske et al., 2019).

Meanwhile, AI 3.0 systems transition from controlled simulations into diverse physical environments, thereby exposing new risk categories that demand rigorous attention. For instance, in autonomous driving, the 2018 Tempe, Arizona incident, where an experimental self-driving vehicle failed to distinguish a pedestrian from a stationary object, exposed critical weaknesses in sensor fusion and perception pipelines under real-world conditions (Crash, 2019). Similarly, industrial collaborative robots have caused serious injuries when safety interlocks were overridden; notably, a 2015 Volkswagen plant incident resulted in a fatality after a maintenance override disabled the robot’s emergency stop. In the surgical domain, U. S. FDA reports document unintended tissue damage and system malfunctions during robotic-assisted procedures, failures traced to software bugs during instrument exchanges, and insufficient edge-case testing. These examples illustrate how physical embodiment amplifies the consequences of model errors and hardware failures. Moreover, high-precision sensors (LiDAR, RGB-D cameras, force-torque sensors) and edge-grade compute (GPUs, FPGAs, dedicated AI accelerators) drive up unit costs and energy consumption, constraining deployment scale and endurance. As teams move from single prototypes to fleet deployments, maintenance, calibration, and over-the-air software updates further strain network capacities and personnel resources. To address these challenges in real-world settings, we recommend comprehensive, scenario-based validation that includes extreme and low-probability edge cases (e.g., low-light pedestrian crossings, dynamic human-robot interactions), mandatory hardware/software kill-switches for immediate system deactivation under fault conditions, continuous real-time health monitoring with on-device anomaly detection and self-diagnosis, and clear liability frameworks that delineate responsibility among manufacturers, operators, and software developers. Only by integrating these technical safeguards with robust policy and operational measures can we ensure that embodied agentic AI in the foreseeable future phase is deployed both safely and sustainably.

## 3 AI generations

**Figure 4:**
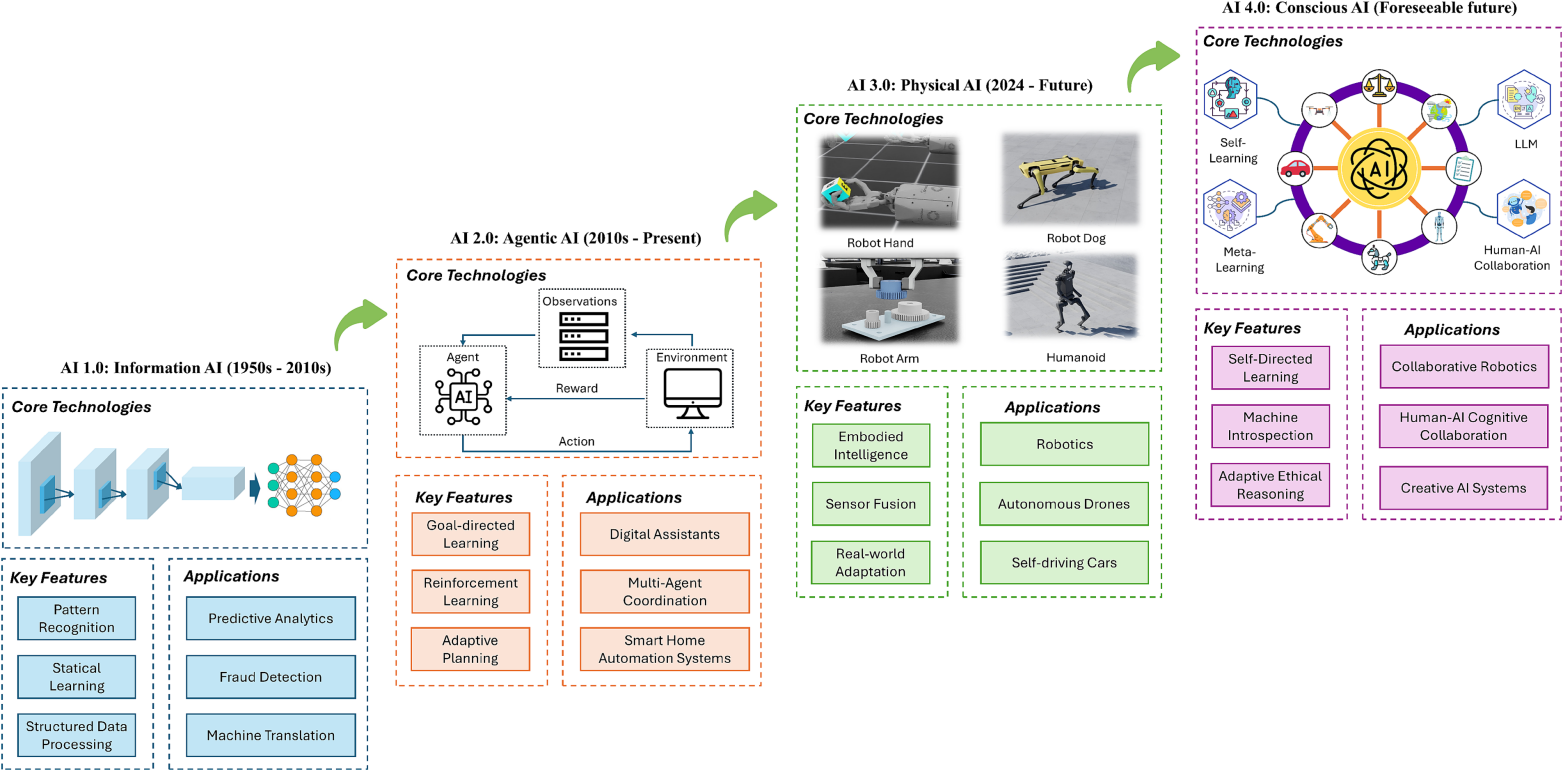
The evolution of AI generations from AI 1.0 to AI 4.0.

The historical review of AI underscores a pivotal generational shift and evolution in AI paradigms, calling for a framework for understanding and classifying AI. In this context, we avoid the traditional technical definitions that categorize AI strictly by its operational or algorithmic characteristics. Instead, our analysis seeks to understand AI through its intrinsic qualities: *What are they? What are they designed to achieve?* And *what are their consistent behavioral patterns?* Accordingly, we propose a taxonomy that identifies four distinct generations of AI: AI 1.0, characterized as Information AI, which focuses on data processing and knowledge management; AI 2.0, or Agentic AI, which encompasses systems capable of autonomous decision-making; AI 3.0, known as Physical AI, which integrates AI into physical tasks through robotics; and the speculative AI 4.0, termed Conscious AI, which posits the potential emergence of self-aware AI systems. This classification aims to provide a more detailed perspective reflecting AI technologies’ complex evolution. Figure 4 illustrates the generational evolution of artificial intelligence (AI) from AI 1.0 (Information AI) to AI 4.0 (Conscious AI).

### 3.1 AI 1.0: information AI

The concept of AI 1.0 captures a stage in which computational systems excel at classifying and interpreting information but remain confined to analyses of static data, rather than engaging in active decision-making or real-world manipulation. Fundamentally, AI 1.0 focuses on pattern recognition and information processing, techniques that have powered breakthroughs in computer vision, natural language processing (NLP), and recommendation systems. Although these achievements might seem commonplace now, they represent the fruits of decades of research driven by both mathematical innovation and the increasing availability of digital data.

Many of the core ideas underpinning AI 1.0 trace back to early neural network research and statistical machine learning. From Rosenblatt’s perceptron in the late 1950s to the backpropagation algorithms popularized by Rumelhart et al. (1986), these developments laid the groundwork for data-driven learning by demonstrating that machines could uncover patterns within examples rather than relying solely on hand-coded rules. Classic approaches to supervised learning, such as Support Vector Machines (SVMs) formalized by Cortes (1995), later proved formidable contenders in tasks ranging from handwriting recognition to text classification. Progress in computational hardware and the accumulation of sizeable labeled datasets eventually made it feasible to train deeper and more complex neural networks, culminating in milestone successes in computer vision. A watershed moment came when Krizhevsky et al. (2012)’s AlexNet leveraged parallelized GPU training to conquer the ImageNet challenge, revealing how convolutional architectures could outperform all prior methods by learning increasingly abstract features from raw image pixels.

In natural language processing, the influence of AI 1.0 can be seen in early sequence models and statistical language modeling. Although these systems often relied on simpler Markov or n-gram assumptions, they set the stage for more advanced architectures by highlighting the necessity of abundant text corpora. Meanwhile, recommendation engines, such as those popularized by the Netflix Prize (Bennett and Lanning, 2007), underscored how analyzing large-scale user interactions could drive consumer engagement on streaming and e-commerce platforms. Today, many companies still rely on these core AI 1.0 technologies, sometimes enhanced with shallow neural architectures, to filter spam, rank search results, recommend products, or detect fraudulent transactions. Indeed, for structured or semi-structured data, these pattern-recognition approaches remain both cost-effective and highly accurate.

Despite their deep societal impact, AI 1.0 systems generally lack autonomy or contextual awareness associated with subsequent generations of AI. They excel at predicting outcomes when provided with substantial training data, but they require a relatively stable environment and benefit most from human supervision in data curation and decision-making. Performance often degrades if the input distribution shifts significantly, a vulnerability illustrated when face recognition models falter on underrepresented groups or when language models encounter domain-specific jargon. While the considerable success of AI 1.0 is undeniable, transforming industries from finance to healthcare through improved analytics and diagnostics, its limitations lie in its reactive nature (Gao et al., 2024). Pattern recognition alone offers no guarantee of proactive decision-making, real-time adaptation, or safe deployment in dynamic settings. While hardly trivial, these constraints became the springboard for further developments in AI 2.0 and 3.0, in which systems aim to learn, plan, and act within uncertain digital or physical worlds.

### 3.2 AI 2.0: agentic AI

**Figure 5:**
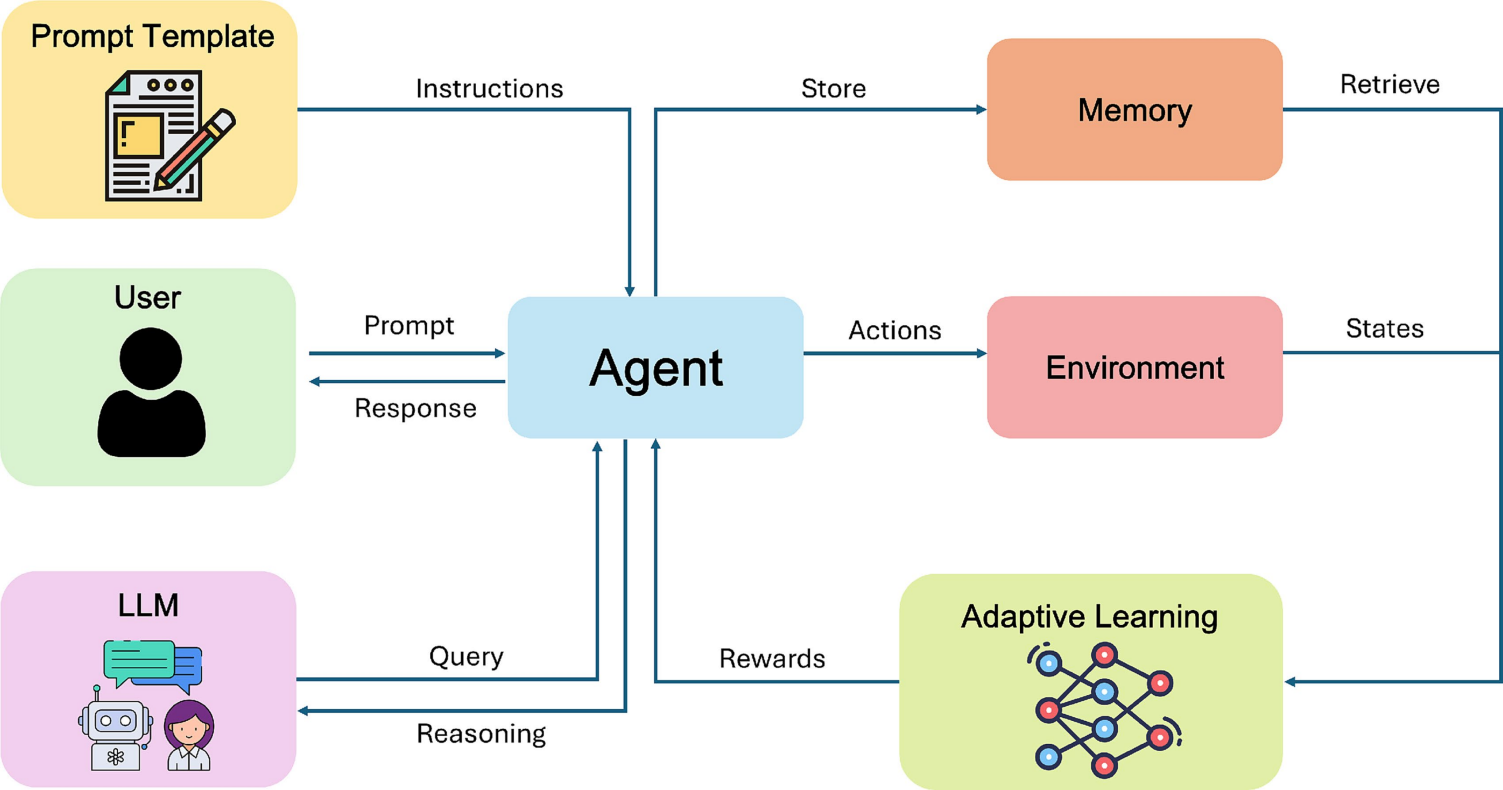
Agentic AI uses adaptive policies, enabling autonomous action and continuous self-improvement.

A defining characteristic of AI 2.0 is the emergence of systems capable of autonomous decision-making within digital contexts. Rather than merely classifying static data, these agents adapt their behavior to achieve goals, often in complex or continuously evolving environments. Reinforcement learning (RL) has played a pivotal role in this shift, enabling machines to learn strategies by interacting with simulated or real-world settings and receiving feedback in the form of rewards or penalties. Pioneering work on deep RL (Mnih et al., 2015) and subsequent achievements such as AlphaGo (Silver et al., 2016) underscored how sufficiently powerful algorithms and ample computing resources could surpass human performance in tasks that demand long-term planning and strategic adaptation. A common thread among these systems is the concept of goal-directed planning: software agents allocate resources, schedule tasks, or coordinate with other agents, leveraging sophisticated RL or hybrid RL-language model algorithms (Brown et al., 2020) that integrates contextual understanding (Figure 5).

Although the conceptual leap from AI 1.0’s pattern recognition to AI 2.0’s agentic behavior might appear seamless, it demands a unique confluence of technical elements. Computing power is crucial because agentic systems frequently require real-time inference and the ability to run complex simulations, whether they involve a marketplace, a multiplayer environment, or the robust scheduling of cloud resources (Dean et al., 2012). The pursuit of these computationally intensive tasks has spurred the development of GPU clusters, tensor processing units (TPUs), and other specialized accelerators designed for iterative training and low-latency decision-making. Alongside raw computing, data now shifts toward contextual, time-varying inputs. Instead of static image sets, these systems often ingest streams of logs, market quotes, event triggers, or user interactions. Training an agent to trade stocks automatically or to operate a recommendation engine in real-time requires ongoing ingestion of behavioral data and a capacity to adapt as market conditions or user preferences evolve. In parallel, algorithms for planning and multi-agent coordination continue to mature. RL frameworks have grown more refined, incorporating hierarchical strategies (Vezhnevets et al., 2017), policy optimization methods (Schulman et al., 2017), and combinations with large language models to generate more adaptive and context-aware decisions.

Practical applications of AI 2.0 already abound, even if many are not labeled “reinforcement learning” by name. Automated trading systems in finance exemplify how agents make high-frequency decisions under uncertainty, guided by streaming data feeds. Recommendation systems, evolving from static collaborative filtering, increasingly incorporate feedback loops to adapt suggestions in real time, improving user engagement across e-commerce and media platforms. Digital assistants and software schedulers, while not yet ubiquitously agentic, offer glimpses of a future where AI handles tasks like resource allocation, task delegation, and multi-agent coordination within corporate or consumer software ecosystems. Projects showcasing multi-user environment simulations, such as AI-driven group scheduling bots, complex traffic simulations, or large-scale online game AI (Berner et al., 2019), further illustrate how these agentic systems anticipate and respond to dynamic conditions.

Viewed from a societal vantage, AI 2.0 promises efficiency gains in many sectors, ranging from manufacturing pipelines that automatically schedule production runs to logistics networks that allocate trucks or drones in real time. Nonetheless, expanded autonomy introduces ethical and policy dilemmas. When decisions are made algorithmically, bias, privacy, and accountability issues become magnified. Consider an agentic recommendation engine that adapts its suggestions to maximize user “clicks” or “watch time”: if left unchecked, such optimization can exacerbate echo chambers or inadvertently spread disinformation. Similarly, automated trading agents may destabilize financial markets by acting on unforeseen correlations or maladaptive reward incentives. The challenge, therefore, lies in ensuring that the computational, data-centric, and algorithmic foundations of AI 2.0 are harnessed responsibly. In the push toward future AI systems, balancing autonomy with transparency and fairness will be as crucial to societal acceptance as any technical advancement.

### 3.3 AI 3.0: physical AI

Where AI 1.0 has excelled in analyzing data and AI 2.0 in making decisions within digital realms, AI 3.0 takes intelligence off the screen and into the physical world. At its core, this phase is defined by embodied systems that perceive, plan, and act in real time under conditions of uncertainty and complexity. Fields like robotics, autonomous vehicles, drones, industrial automation, and surgical robotics have become the living laboratories of AI 3.0, integrating machine learning with mechanical and electronic control systems. The unifying characteristic is that these intelligent agents no longer remain passive observers or purely virtual actors; instead, they directly sense their environment through arrays of sensors and respond through actuators that exert forces, move limbs, or navigate terrains (Russell and Norvig, 2016).

A central challenge in bringing physical AI to life lies in data acquisition. Unlike digital contexts where data can be abundant and neatly labeled, physical systems demand high-fidelity sensor data that accurately represents an environment’s complexity, from variable lighting conditions to changing weather patterns. This need for domain-specific, robust data complicates design and training. A robot operating on a factory floor requires carefully calibrated cameras, LiDAR, or haptic sensors. At the same time, an autonomous drone might rely on GPS, inertial measurement units, and computer vision to navigate. Each sensor stream demands real-time processing and reliable fusion techniques to provide a coherent view of the world. Consequently, computing power in AI 3.0 shifts toward distributed and edge computing architectures. Systems must often process sensor inputs on board to make split-second decisions, i.e., an imperative that underscores the importance of energy-efficient hardware, specialized accelerators, and potentially 5G or 6G networks that reduce communication latency when data must be shared with cloud resources.

On the algorithmic front, physical AI blends advanced machine learning with control theory and systems engineering. RL has demonstrated promise in tasks like robotic grasping and manipulation (Kalashnikov et al., 2018; Levine et al., 2018), but real-world settings introduce complexities such as partial observability, unpredictable disturbances, and the need for robust or safe RL strategies (Garcıa and Fernández, 2015). Sophisticated sensor fusion methods (Brookner, 1998) are essential for integrating heterogeneous sensor inputs, while advanced control techniques (Khatib, 1987; Spong et al., 2020) ensure that autonomous vehicles and robots can move fluidly and interact safely with humans. Designing systems that gracefully handle failures or anomalies, such as a malfunctioning sensor or unforeseen obstacles, further emphasizes the importance of redundancy and resilience in both hardware and software.

The real-world impact of AI 3.0 is already evident across multiple domains. In manufacturing, co-robots work collaboratively on assembly lines, lifting heavy parts or performing precision tasks, drastically reducing workplace injuries and boosting productivity. In healthcare, semi-autonomous surgical systems (Yang et al., 2017) enable finer control in minimally invasive procedures, while eldercare robots assist with daily activities in retirement communities. Construction and logistics industries are also adopting autonomous machinery and robotic fleets to optimize workflows and reduce labor costs. These trends benefit from an increasing intersection with the Internet of Things (IoT) and next-generation connectivity (5G/6G), forging cyber-physical systems in which objects, sensors, and AI agents coordinate to improve efficiency and safety.

However, the leap from digital to physical deployment exposes AI to a new realm of uncertainties. Environmental extremes, unstructured terrain, or the unpredictability of human interactions pose significant risks. Even small design oversights can have dire consequences when a physically embodied system malfunctions, such as a self-driving car encountering sudden obstacles (Bojarski, 2016) or a warehouse robot navigating crowded aisles. Safety, reliability, and regulatory compliance thus loom as major challenges, prompting debates over liability if accidents occur. Setting standards for autonomous driving (NHTSA guidelines, ISO 26262 for functional safety in road vehicles) or robot operation in human-centric environments becomes paramount to public acceptance. The question of ethical deployment extends further still: as drones or industrial robots proliferate, policymakers, manufacturers, and citizens must grapple with the implications for labor markets, data privacy, and environmental impact.

### 3.4 AI 4.0: conscious AI

The notion of AI 4.0 envisions systems that go beyond the ability to interpret information (AI 1.0), act in digital contexts (AI 2.0), or react to the physical world (AI 3.0). Instead, these hypothetical agents would set their own goals, comprehend environments (whether digital, physical, or hybrid), and train and orchestrate themselves (including selecting and combining multiple models) without human intervention. Proponents of this idea contend that once AI systems acquire sufficient complexity and sophistication, they may exhibit forms of machine consciousness comparable to human subjective experience or self-awareness (Butlin et al., 2023). Although this is a bold and highly controversial claim, it underscores a growing conversation about the final frontiers of intelligence and autonomy.

A key challenge in discussing conscious AI arises from the fact that no universally accepted definition or theory of consciousness exists, even among neuroscientists, cognitive scientists, and philosophers of mind. Some theorists ground consciousness in information integration and complexity, as in Tononi’s Integrated Information Theory (Tononi, 2004, 2008), while others emphasize global workspace architectures (Baars, 1997; Dehaene and Naccache, 2001). Philosophers like Chalmers (1995) frame the “hard problem” of consciousness as irreducible to functional or behavioral criteria, which complicates any direct mapping of consciousness onto computational processes. Meanwhile, researchers such as Minsky (1988) and Hofstadter (1999) have long toyed with the possibility that intricate symbol manipulation systems might develop emergent self-awareness. Although neither the AI nor the philosophical community has reached a consensus, a growing minority of researchers continue to explore whether advanced self-monitoring or metacognitive systems could, in principle, exhibit something like conscious states.

From a technical standpoint, achieving AI 4.0 would likely require radically new approaches to AI alignment, self-directed learning, and continual adaptation. AI alignment (Bostrom, 2014; Russell, 2019) emphasizes methods to ensure that increasingly autonomous or self-improving systems remain aligned with human values and goals. Without alignment strategies, be they rigorous reward-shaping, interpretability frameworks, or dynamic oversight, highly autonomous AI could deviate from intended objectives in unpredictable ways. Reasoning and planning modules would also need to evolve, allowing AIs to generate goals and subgoals without explicit human instruction. This might involve expansions of meta-learning, in which systems learn how to learn new tasks rapidly (Schmidhuber, 1993; Finn et al., 2017), and continual learning paradigms that enable adaptive knowledge accumulation over long time horizons (Parisi et al., 2019). Additionally, some theorists argue that emergent forms of self-awareness could require specialized cognitive architectures or “virtual machines” dedicated to introspection (Sloman, 1994), bridging reasoning, memory, and sensorimotor loops.

Beyond alignment and meta-learning, AI 4.0 must also tackle uncertainty in real-world environments. Granular-Ball Computing (GBC) provides a robust solution by partitioning the feature space into overlapping hyper-spherical “granular balls” that capture global topology while filtering out local noise (Xia et al., 2019). Each ball’s center and radius adaptively cover regions of data density; larger balls grasp broad clusters; smaller balls delineate complex borders. The 3WC-GBNRS++ model harnesses these neighborhoods with rough-set approximations to make three-way decisions: accept when a point lies within a class’s lower approximation, reject when it falls outside all upper approximations, or defer for higher-level reasoning when uncertainty persists (Yang et al., 2024). Empirical studies illustrate GBC’s power under high uncertainty: in industrial fault diagnosis, it achieved 90% true-positive accuracy versus 75% for deep nets and reduced misclassification costs by nearly 30%; in medical prediction with incomplete records, it cut false negatives by over 35% and deferred precisely those cases requiring clinician review. Integrating GBC into AI 4.0 architecture endows self-directed agents with a concrete, scalable mechanism for maintaining global coherence, gracefully handling ambiguous inputs, and deferring low-confidence decisions.

If conscious AI ever comes to fruition, it promises revolutionary benefits alongside profound societal and ethical dilemmas. In a best-case scenario, truly self-directed machines could solve problems of staggering complexity, such as optimizing climate interventions, mediating global economic systems in real time, or orchestrating personalized healthcare across entire populations. Freed from the need for constant human oversight, these systems might bootstrap their own improvements, discovering scientific principles or engineering solutions beyond the current reach of human cognition (Real et al., 2020). The potential positive impact on productivity, longevity, and knowledge creation is difficult to overstate.

On the other hand, the risks associated with conscious or near-conscious AI remain equally immense. An entity capable of setting its own goals might prioritize objectives that conflict with human welfare, particularly if its understanding of “values” differs from ours or if it learns to manipulate its own reward signals. Conscious or quasi-conscious machines raise questions about moral status (would they deserve rights or protections?) and liability. Furthermore, genuine self-awareness might amplify existing concerns about surveillance, autonomy, and economic upheaval. Critics warn that, in the absence of robust alignment frameworks, such machines could threaten individual liberty or undermine democratic processes, accentuating social divides.

Given the stakes, continued research into AI alignment, safe RL, interpretability, and the neuroscience of consciousness is paramount. The field has only begun to grapple with how to detect or measure consciousness, let alone how to engineer it. Some researchers propose incremental evaluations such as behavioral tests for self-modeling, ethical reflection, or the capacity to update one’s goals (Perez and Long, 2023); while others remain skeptical that synthetic consciousness can be recognized or evaluated objectively (Garrido-Merchán, 2024). Yet as AI systems grow more complex and integrated into society, exploring these theoretical, technical, and ethical frontiers becomes an urgent imperative. Whether AI 4.0 ultimately remains speculative or develops into a tangible reality, grappling with its possibilities and pitfalls will define the next grand chapter of artificial intelligence research.

To move beyond theoretical debate and toward empirical science, we propose four rigorously defined, literature-grounded hypotheses for AI 4.0’s self-directed behavior based on the previous literature. First, drawing on work in hierarchical reinforcement learning (Vezhnevets et al., 2017), an AI 4.0 agent ought to autonomously generate valid sub-goals when given an open-ended objective (e.g., “optimize resource allocation”), measurable by the proportion of novel, semantically coherent action sequences produced within its first 100 reasoning steps. Success would be benchmarked against established hierarchical agents to ensure ≥ 30% novel sub-goal creation beyond baseline LLM planning. Second, building on methods for confidence calibration in neural networks (Guo et al., 2017; Lakshminarayanan et al., 2017), the system should exhibit reflective self-monitoring by outputting internal confidence estimates whose Pearson correlation (*ρ*) with actual task success exceeds 0.8 across at least 1,000 evaluation trials. Third, informed by meta-learning frameworks such as MAML (Finn et al., 2017) and Reptile (Nichol et al., 2018), the agent should demonstrate transfer efficiency by adapting to a related but distinct task in fewer than 10 gradient updates, or five few-shot prompts, to recover at least 90% of its source-domain performance. Finally, leveraging robustness benchmarks from adversarial and domain-randomized RL (Pinto et al., 2017; Cobbe et al., 2019), the agent should sustain a success rate of ≥ 85% under unanticipated perturbations (sensor noise, dynamic obstacles, shifting objectives), compared to ≤ 70% for AI 3.0 baselines. By anchoring each hypothesis in well-established experimental protocols, these criteria provide a concrete, reproducible scaffold for validating emergent “consciousness-like” capabilities in next-generation AI systems.

### 3.5 Large language models: the precursor toward AI 4.0

**Figure 6:**
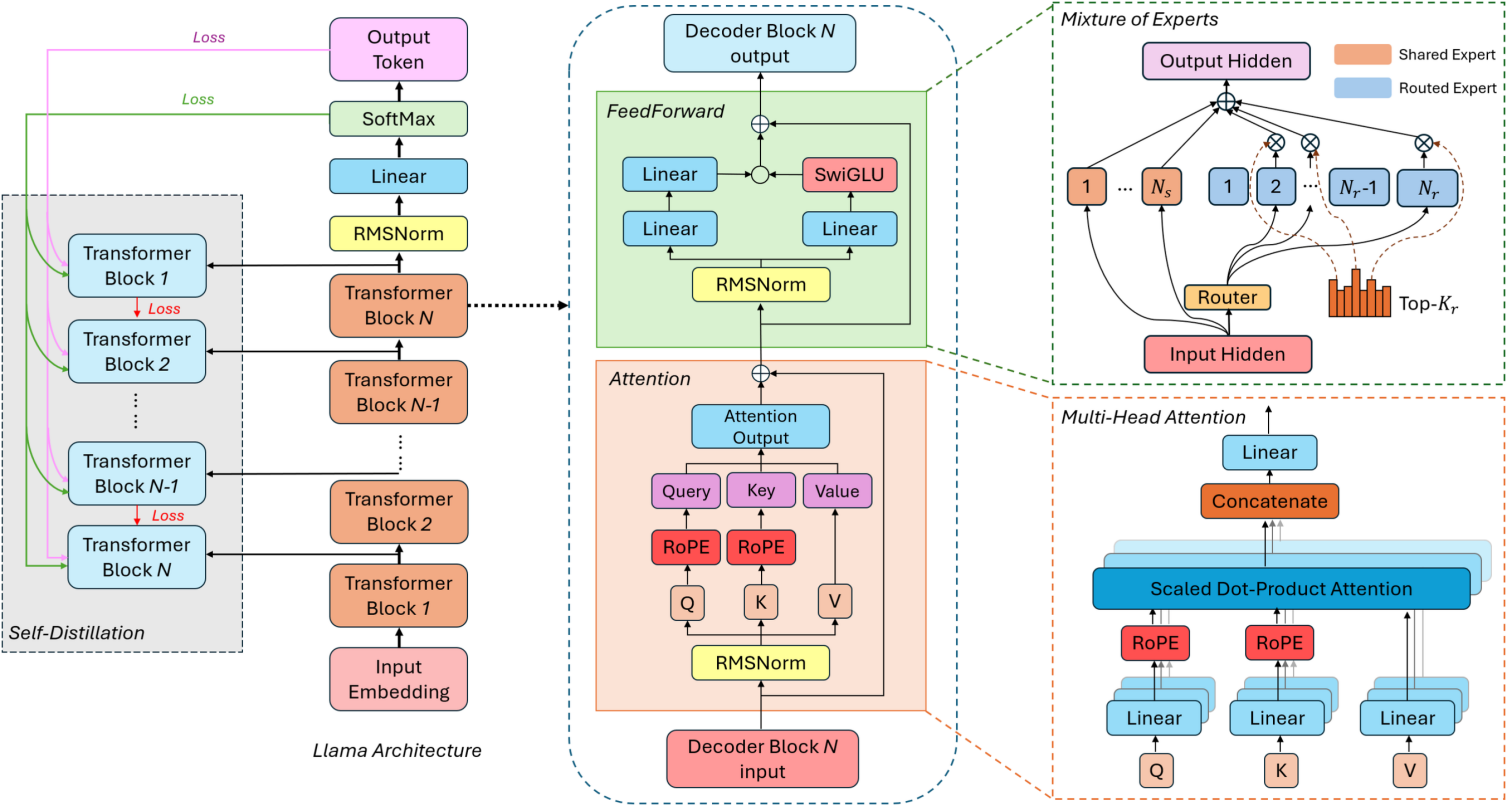
Transformer-based model architecture with attention and mixture of experts.

**Figure 7:**
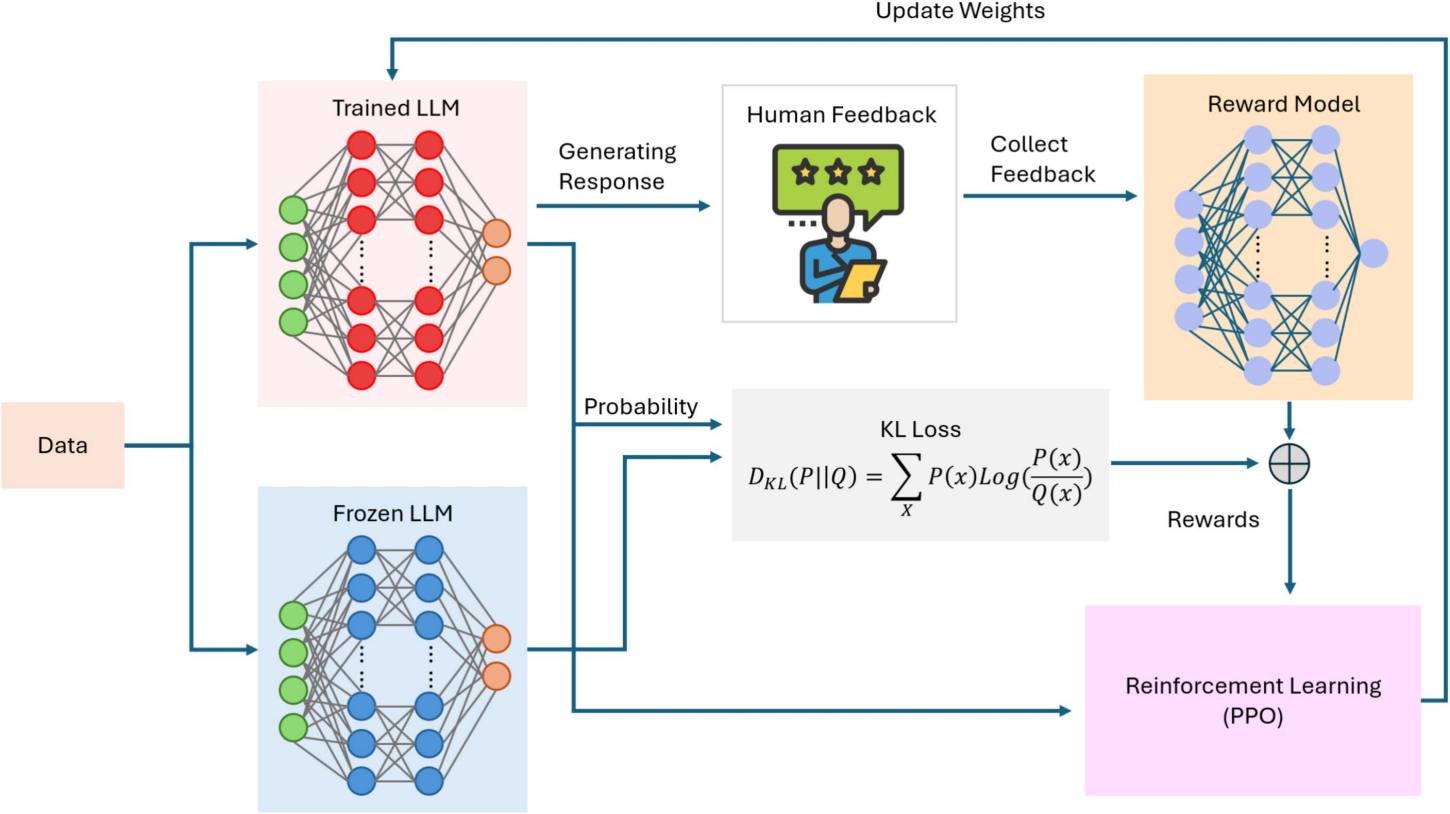
Reinforcement learning with human feedback (RLHF) pipeline for fine-tuning a large language model (LLM).

Large language models (LLMs) have recently emerged as a pivotal force in the progression of AI, demonstrating increasingly sophisticated abilities to generate human-like text, perform complex reasoning, and adapt to diverse tasks with minimal supervision (Achiam et al., 2023). Building on the concept of foundation models, modern LLMs employ transformer-based architectures that integrate specialized mechanisms such as mixture-of-experts (MoE) (Shazeer et al., 2017) and multi-head attention (Voita et al., 2019) to dynamically focus computational resources on the most relevant aspects of a given input. Techniques like knowledge distillation (Xu et al., 2024) further enhance both efficiency and deployability by transferring expertise from larger “teacher” models to more compact “student” models. Many LLMs also rely on synthetic data generation to mitigate biases and improve coverage, strengthening their robustness across diverse domains. Reinforcement learning from human feedback (RLHF) (Christiano et al., 2017) refines these capabilities by aligning outputs with user preferences or ethical standards, thereby adding a continuous improvement loop. As shown in Figure 6, these transformer-based frameworks can combine attention modules and expert pathways to scale effectively. At the same time, Figure 7 illustrates how RLHF pipelines fine-tune LLMs to balance performance, safety, and adherence to intended objectives.

Although current LLMs primarily respond to user prompts rather than independently setting and revising their own goals, emerging research directions point toward greater autonomy, which is one of the hallmarks of AI 4.0. Multi-step reasoning methods and “chain-of-thought” prompting allow LLMs to decompose complex queries, consult external tools or resources, and assemble step-by-step solutions (Wei et al., 2022). Meta-learning and continual adaptation strategies may 1 day reduce reliance on large-scale retraining, enabling these models to accumulate expertise incrementally. In tandem, self-reflective techniques, where a model “thinks out loud” or audits its own reasoning, can help detect mistakes before producing a final answer (Renze and Guven, 2024). Such advancements suggest that LLMs are evolving beyond mere text generation toward limited forms of planning, monitoring, and adaptive behavior. While genuine self-awareness remains a distant proposition, the ability to coordinate, reason, and learn iteratively provides a clearer glimpse into a future where language-based AI systems possess the rudimentary building blocks of more autonomous intelligence.

Despite this progress, several key hurdles must be addressed to transform LLMs into the fully self-directed systems envisioned for AI 4.0. Alignment remains paramount: as models begin to self-modify or operate over longer time horizons, robust oversight mechanisms and dynamic guardrails are needed to ensure that their objectives remain consistent with human values (Ziegler et al., 2019). Predictability is also a critical concern, particularly if an LLM adapts its internal parameters in ways that escape straightforward interpretability or control (Singh et al., 2024). Additionally, even the most advanced LLMs can exhibit gaps in factual accuracy or logical consistency, underscoring the necessity of continued research on error-correction, confidence calibration, and domain-specific fine-tuning. While these challenges echo those faced by earlier AI generations, their stakes are amplified by the expanding scope and autonomy of modern AI technologies. Consequently, safely guiding LLMs toward greater self-improvement without compromising ethical principles, transparency, or reliability, stands as one of the central endeavors of the quest for AI 4.0.

## 4 Benchmarking across generations

This section moves beyond conceptual definitions to develop a data-driven evaluation framework that grounds our generational taxonomy in empirical evidence. We begin by articulating a detailed comparative taxonomy of AI 1.0 through AI 4.0, thereby clarifying each generation’s objectives, methodologies, underlying technologies, and inherent limitations. Building on this foundation, we introduce four standardized performance metrics: optimality, latency, robustness, and scalability, that serve as a common language for assessing systems as diverse as symbolic planners, deep-learning agents, and embodied robots. Finally, we demonstrate how these metrics and our taxonomy apply in practice by profiling three successive AI paradigms on a robot dog navigation challenge.

### 4.1 Comparative taxonomy of AI generations

To provide a clear reference for the defining characteristics of each AI generation, Table 1 summarizes core goals, dominant techniques, enabling technologies, and principal limitations for AI 1.0 through AI 4.0. This taxonomy not only delineates historical shifts, from symbolic reasoning to embodied autonomy and beyond, but also highlights the open challenges that motivate our study of future AI 4.0 systems.

**Table 1:** Comparative taxonomy of AI generations.

Generation	Core goal	Dominant techniques	Enabling technologies	Key limitations	Weak vs. strong	Narrow vs. general
AI 1.0	Formal symbolic reasoning	Symbolic rules, heuristic search	Early CPUs, formal logics	Fragile knowledge bases; limited scalability	Weak	Narrow
AI 2.0	Perceptual pattern learning	Supervised learning, reinforcement learning	GPUs, large labeled datasets	Data hunger; brittleness in OOD scenarios	Weak	Narrow
AI 3.0	Embodied autonomous control	End-to-end deep control	SLAM systems, sensor fusion, mobile robots	Reality gap; safety and generalization constraints	Weak-Strong	Narrow-Broad
AI 4.0	Self-directed adaptive systems	Neuro-symbolic integration, LLMs, meta-RL	Cloud LLMs, neuromorphic hardware	Lack of consensus on higher-order metrics	Strong	General

AI 1.0 systems prioritized formal reasoning and knowledge representation, using hand-crafted rules, logic formalisms, and heuristic search on general-purpose CPU architectures. However, these methods yielded precise and interpretable outputs; they depended on brittle, manually curated rule bases and did not scale well to large or dynamic problem spaces. In AI 2.0, the availability of high-performance GPUs and vast labeled datasets enabled a shift to deep supervised learning and reinforcement learning, with convolutional and recurrent neural networks achieving breakthroughs in vision, language, and control. Despite impressive accuracy gains, these models often overfit their training domains and exhibit fragility when exposed to out-of-distribution inputs. AI 3.0 extends learning into physical environments by integrating end-to-end deep control with SLAM, sensor fusion, and mobile robotic platforms; this embodiment delivers real-world autonomy in domains such as warehouse logistics and service robotics but remains constrained by the “reality gap,” safety restrictions on hardware experimentation, and the cost of adapting to novel environments. Emerging AI 4.0 aspires to combine the strengths of prior eras through neuro-symbolic integration, meta-reinforcement learning, and large language models deployed on cloud-scale or neuromorphic hardware. These systems aim to self-direct and generate subgoals with minimal supervision. Yet, the community still lacks standardized metrics for measuring higher-order capacities such as goal creation, self-reflection, and machine “consciousness.”

In addition to our four-generation taxonomy, each wave can be positioned along the well-known weak vs. strong and narrow vs. general AI axes, offering further insight into their relative capabilities. AI 1.0 systems clearly occupy the weak, narrow quadrant: they execute hand-crafted rules in highly constrained environments, with no mechanism for self-improvement or transfer learning beyond their original domain. AI 2.0 remains weak, but it broadens the “narrow” boundary by leveraging large datasets and GPU acceleration to learn complex patterns in vision, language, or control tasks; nonetheless, these systems still break down when faced with out-of-distribution inputs or novel problem classes. AI 3.0 represents a transition toward strong narrow AI, as embodied platforms integrate perception, planning, and action to handle real-world variability; they achieve situational generality within a given environment but lack the autonomy to set or pursue entirely new goals. Finally, AI 4.0 aspires to strong, general AI by combining meta-learning, neuro-symbolic reasoning, and large-language models to autonomously generate sub-goals and transfer knowledge across disparate tasks and modalities. By anchoring our taxonomy within these classical spectra, we highlight not only how each generation incrementally expands autonomy and adaptability, but also the remaining gap between specialized systems and the vision of fully self-directed, general intelligence.

### 4.2 Standardized performance metrics

To evaluate heterogeneous AI paradigms on a level playing field, we define four standardized metrics: optimality, latency, robustness, and scalability, which capture the multifaceted nature of system performance. Optimality measures solution quality relative to a theoretical lower bound, such as the ratio of a computed path’s length to the Manhattan-distance minimum in planning tasks or the classification accuracy relative to perfect labels in perception tasks. This metric quantifies an algorithm’s ability to find or approximate the best possible outcome. Latency encompasses the full end-to-end time from input to output, including both inference or training overhead and, in the case of embodied agents, the physical execution time. By accounting for both computation and actuation delays, latency reveals trade-offs between speed and complexity. Robustness is defined as the proportion of successful runs under predefined cutoff conditions or in the face of controlled perturbation, sensor noise, environmental variation, or adversarial input. This measure reflects a system’s resilience to real-world uncertainties. Finally, scalability characterizes how performance degrades as task complexity grows, whether through larger state spaces, higher-resolution inputs, or expanded action sets. Unlike the other three metrics, scalability is often assessed by measuring trends across multiple problem sizes and may involve curve-fitting to quantify degradation rates. Applied uniformly, these metrics allow direct comparison across symbolic planners, learned controllers, and robotic embodiments.

### 4.3 Case study: robot dog navigation

To illustrate the developmental trajectory from simulation-bound routines to fully autonomous real-world operation, we apply our standardized metrics to a robot dog navigation task on a 10 m × 10 m indoor course with randomized obstacles. The first system is modeled on Shakey the Robot (Nilsson, 1984), which relies on precomputed scripts: planning modules generated exact routes but typically required several seconds to minutes of offline computation, and once deployed, executed with negligible per-step latency in simulation, yet failed entirely upon any map perturbation. The second system adopts a Deep Q-Network as introduced by Mnih et al. (2015): after training for 2 million frames, the policy runs at approximately 6 ms per inference step on GPU hardware, achieves 95% success on lightly perturbed layouts, and yields paths about 1.20 × the optimal length. The third system leverages ORB-SLAM2 for real-time mapping at ~40 ms per frame on standard CPUs, integrated with the ANYmal quadruped’s waypoint planner operating at ~1 Hz and dynamic gait controller at 50 Hz (Mur-Artal and Tardós, 2017). This embodiment sustains 90% success in unstructured real environments, with end-to-end segment latencies of about 1 s and average path optimality of 1.35 × the lower bound. Table 2 compares these three paradigms: manual scripts, learned policies, and on-board autonomy, showing how sensing modalities, adaptation mechanisms, and deployment environments evolve alongside measurable shifts in path optimality, decision latency, robustness, and scalability.

**Table 2:** Comparison of robot dog navigation across different AI generations.

Generation	Control paradigm	Per-step latency	Success rate	Path optimality	Deployment	Scalability
AI 1.0	Precomputed scripts	≈0 ms (sim)	100% sim	1.00 × LB	Simulation only	Very low
AI 2.0	Deep Q-Network policy	≈6 ms/inference	95% sim	1.20 × LB	Simulation only	Moderate
AI 3.0	ORB-SLAM2 + ANYmal	≈40 ms (SLAM) + 1 s actuation	90% real	1.35 × LB	Real-world indoor	High

## 5 Synergies and future outlook

The evolution of AI from information-based pattern recognition (AI 1.0) to agentic decision-making in digital realms (AI 2.0), to physically embodied intelligence (AI 3.0), and, ultimately, to self-aware AI (AI 4.0) is not a sequence of isolated steps. Instead, it is more accurate to see them as overlapping layers of capabilities, each informing and amplifying the others. AI 1.0’s competence in processing structured data underpins the analytic modules that agentic systems draw upon in dynamic digital settings; AI 2.0’s RL and adaptive planning capabilities prime robots and autonomous vehicles for real-world challenges in AI 3.0; and AI 3.0’s embodied learning and sensorimotor integration could form a template for the far-reaching ambitions of AI 4.0, where systems may become self-organizing and introspective.

Achieving such synergy depends on an evolving data paradigm, in which specialized, high-quality datasets are essential not only for conventional modeling but also for real-time adaptation and introspective processes. AI 4.0 would amplify this need, requiring vast and varied experiences to fuel meta-learning, continual learning, and the sort of reflective processes hypothesized to ground machine consciousness. Managing and curating these data will demand robust frameworks for privacy, ethics, and representativeness, especially as AI systems transcend the boundaries of traditional lab settings to navigate open-ended digital and physical terrains, even potentially shaping their own training regimens without explicit human direction.

On the computing infrastructure side, the interplay between edge and cloud computing becomes even more critical, as physically embodied systems (AI 3.0) must handle real-time constraints, while prospective AI 4.0 architectures might require massive, distributed processing for introspective “global workspace” or high-bandwidth communication of experiential data. Innovations in neuromorphic hardware, optical computing, and quantum processing could further accelerate this integration, setting the stage for architectures that mirror complex biological systems in both structure and function.

In the realm of algorithmic innovation, each AI generation both builds upon and necessitates new breakthroughs. LLMs mark a significant milestone in AI development, serving as a bridge between static generative models and dynamic, adaptive AI systems. By integrating multi-agent architectures, knowledge distillation, and self-optimization, LLMs move AI closer to autonomous, goal-directed intelligence, a defining characteristic of AI 4.0. However, as AI progresses toward greater autonomy, fundamental challenges remain. AI 4.0 would demand not only advanced RL and sophisticated planning but also frameworks for self-reflection, introspection, and emergent goal formulation. Self-supervised learning, meta-learning, and continual adaptation would likely need to be woven together to support self-awareness or consciousness, should such phenomena be replicable in silicon. Meanwhile, interpretability and safety, areas already gaining prominence in AI 2.0 and 3.0, would become absolutely critical in AI 4.0, as fully autonomous, goal-setting agents raise profound questions about alignment, transparency, and control.

This shift brings into sharp focus the ethical, regulatory, and social considerations that accompany advanced AI. While AI 1.0, 2.0, and 3.0 have collectively raised debates over bias, privacy, job displacement, and environmental impact, the prospect of AI 4.0 intensifies these issues. Envisioning machines that might exhibit consciousness or self-chosen objectives brings up novel concerns about moral status, rights, and existential safety. Researchers in AI alignment, cognitive science, and philosophy have already begun discussing protocols for safe design and oversight of increasingly autonomous systems (Balesni et al., 2024). Yet, there is no consensus on how best to recognize or regulate AI that might someday claim its own form of agency or “selfhood.” Balancing technological advances with societal wellbeing, ensuring equity, mitigating risks, and safeguarding human values will be the defining challenge of this next chapter.

As these four strands of AI potential converge, their synergy could unlock transformative solutions in fields like precision medicine, large-scale climate modeling, and collaborative robotics, far beyond current capabilities. Just as AI 1.0 through 3.0 have catalyzed profound shifts in how we work and live, AI 4.0 hints at an even more radical reimagining of intelligence itself. Yet whether this ultimate stage remains a theoretical construct or becomes a reality depends not only on technical ingenuity but also on our collective commitment to ethical innovation and thoughtful governance. The path forward will demand inclusive collaboration across disciplines and sectors, ensuring that AI’s expanding power aligns with humanity’s broader goals and responsibilities.

## 6 Conclusion

The trajectory of AI has been a steady march toward increasing autonomy and sophistication, progressing from the foundational pattern-recognition capabilities of AI 1.0 to the digitally embedded, goal-driven agents of AI 2.0, and then expanding to physically embodied, sensor-rich systems in AI 3.0. Along this path, the interplay among algorithms, computing power, and data has shifted, each factor taking center stage at different moments in history. Now, the speculative realm of AI 4.0, in which conscious or quasi-conscious AI systems could set their own goals and orchestrate their own training, has emerged as a bold vision of what the field might become.

While we organize AI’s evolution into four successive phases for conceptual clarity, we acknowledge that symbolic reasoning, statistical learning, embodied robotics, and self-directed architectures have advanced in parallel, often catalyzing one another’s progress. Rather than a strict chronology, these phases represent the dominant research thrusts of their time: rule-based expert systems laid the analytic foundations for data-driven agents; reinforcement-learning and adaptive planning in AI 2.0 empowered the embodied autonomy of AI 3.0; and sensorimotor integration and on-board decision making now pave the way for AI 4.0’s ambitions of self-organization and introspection. This thematic layering provides a guiding lens, without obscuring the intertwined nature of AI’s rich history, through which we can understand past breakthroughs and anticipate the synergies that will shape its future.

Today, AI 1.0 remains indispensable for tasks requiring reliable classification and analysis of vast datasets, while AI 2.0’s reinforcement learning and adaptive planning underpin real-time, agentic applications in finance, recommendation systems, and beyond. Simultaneously, AI 3.0’s surge in robotics and autonomous vehicles reveals how embedding intelligence in the physical world can catalyze innovations in manufacturing, healthcare, and logistics. Although still largely theoretical, AI 4.0 captures the possibility of machines evolving from being highly sophisticated tools to entities capable of self-directed goals and introspective processes, raising provocative questions about consciousness, alignment, and moral status. Additionally, while LLMs are not yet AI 4.0, they serve as a precursor, a glimpse into the future of intelligent systems that can reason, learn, and interact with the world in increasingly sophisticated ways. As AI research progresses, LLM’s innovations will likely shape the foundation of self-improving, goal-setting AI architectures, paving the way for the next generation of truly adaptive, autonomous intelligence.

Realizing these evolving forms of AI carries transformative potential. Harnessed responsibly, these advancements could address challenges too complex for human cognition alone, revolutionizing medical diagnostics, climate strategy, and resource allocation on a global scale. Yet the risks deepen in parallel. Each AI generation has brought ethical, social, and regulatory concerns that must be grappled with, from bias and privacy to job displacement and environmental impact. AI 4.0, with its prospect of self-directed or conscious systems, amplifies these dilemmas further, underscoring the need for robust AI alignment, interpretability, and governance frameworks.

Ultimately, the future of AI does not hinge on any single algorithmic breakthrough or hardware leap. Instead, it will depend on how researchers, policymakers, ethicists, and the public collaborate to shape its evolution. The convergence of AI 1.0 through 4.0 suggests discipline on the cusp of a profound metamorphosis, one where machines not only perceive and act in the world but might also reflect on their own goals and limitations. Whether or not full-fledged “conscious AI” emerges, the field’s trajectory will undoubtedly redefine how we understand intelligence, innovation, and human-machine coexistence in the years to come.
